# eHMI: Review and Guidelines for Deployment on Autonomous Vehicles

**DOI:** 10.3390/s21092912

**Published:** 2021-04-21

**Authors:** Juan Carmona, Carlos Guindel, Fernando Garcia, Arturo de la Escalera

**Affiliations:** Intelligent Systems Lab, Universidad Carlos III de Madrid, 28903 Madrid, Spain; cguindel@ing.uc3m.es (C.G.); fegarcia@ing.uc3m.es (F.G.); escalera@ing.uc3m.es (A.d.l.E.)

**Keywords:** human–machine interface, external human–machine interface, autonomous vehicles, interaction pedestrian–vehicle

## Abstract

Human–machine interaction is an active area of research due to the rapid development of autonomous systems and the need for communication. This review provides further insight into the specific issue of the information flow between pedestrians and automated vehicles by evaluating recent advances in external human–machine interfaces (eHMI), which enable the transmission of state and intent information from the vehicle to the rest of the traffic participants. Recent developments will be explored and studies analyzing their effectiveness based on pedestrian feedback data will be presented and contextualized. As a result, we aim to draw a broad perspective on the current status and recent techniques for eHMI and some guidelines that will encourage future research and development of these systems.

## 1. Introduction

User interfaces are an essential part of any complex system that requires communication with the user. ISO 9241-110 [1] defines them formally as “all components of an interactive system (software or hardware) that provide information and controls for the user to accomplish specific tasks with the interactive system.” A user interface is composed, in turn, of a human–machine interface (HMI) (also referred to as man–machine interface, MMI), which is responsible for establishing physical communication between both counterparts (i.e., user and system). Thanks to this, the user can observe the status of the system but also act on it, modifying the parameters of its operation. Information (“feedback”) is provided by control panels with light signals, display fields or buttons, or by software using a display system running on a terminal, for example.

Human–machine interaction is an active field of research nowadays, and its importance is expected to grow even more in the next decades. Advances in the matter have made it possible for users to interact with all kinds of devices naturally, confidently, and correctly, in both personal and professional environments. Different disciplines are involved in this topic, such as engineering, cognitive research, humanities, and psychology. The basic knowledge for an interface design that is easy for the user to use is gathered in the scientific discipline of ergonomics.

The success of a technical product depends on more factors than just price, reliability, and life cycle; it also depends on factors such as handling capacity and ease of use. Therefore, HMIs are a crucial aspect in the design of devices that involve interaction with a person.

Generally speaking, a robust HMI must be multifaceted, speedy in providing a response, cost-effective, adaptable to the environment, and easy to understand [2]. However, as an increasing fraction of society interacts with technology, the design must consider a wide variety of users, including children, elders, people with disabilities, and, in general, people with very diverse technological skills. This diversity explains, in part, the enormous number of interface types that exist today. 

One of the areas with the most remarkable growth and relevance in the development of HMIs is the automotive field. Good machine–user (i.e., vehicle–driver) communication is particularly crucial in a field where different decisions are made in a short time [3]. Ideally, a human–machine interface (HMI) would be explained intuitively, without the need for training; this simple premise is even more important when dealing with HMIs in vehicles.

Thanks to the development of the automotive industry in the last few decades, modern vehicles are faster, more efficient, and more comfortable than ever. They are already equipped with most of the components that will integrate future autonomous vehicles: elements such as sensors or processing units are already available to assist the driver. Autonomous driving aims to further progress in road safety, given that, according to the European Commission [4], 90% of all serious car accidents are due to factors related to human error, such as reckless driving, distraction, speeding, or illegal maneuvering. 

The changes in automation and connectivity represent an opportunity to move towards a more efficient, safer, and less polluting transportation paradigm, which will likely enhance accessibility by pushing changes in the current vehicle ownership model (e.g., car-sharing) [5]. Predictably, the advantages of this technology will be apparent to the general population shortly after its introduction; however, as it involves profound changes in the way people understand mobility, some reluctance is to be expected, especially if the behavior of AVs is not well understood by the different groups of road participants, or reliability issues arise. These circumstances could significantly slow down the deployment of AV technology.

Hence, among the different tasks that must be carried by a vehicle to realize autonomous driving, the interaction with other traffic participants is one of the most critical. In particular, vulnerable road users (VRUs), namely pedestrians, cyclists, and motorcyclists, pose a significant risk for autonomous navigation when their trajectories converge with the one followed by the vehicle. 

A great deal of effort has been devoted in recent years to the identification of these agents so that vehicles can be aware of their presence in advance and make decisions accordingly. Systems used to that end are typically based on the information from on-board sensors [6], although broader cooperative clusters including communication with external nodes(e.g., unmanned aerial vehicles [7,8]) are to be expected in the near future. Nonetheless, mechanisms to make VRUs aware of the vehicle’s awareness are usually not foreseen. 

HMIs are used profusely in today’s vehicles to allow the driver (and the other occupants) to interact with the vehicle. However, communication with other road users (both other drivers and VRUs) has attracted much less interest. Only recently, car manufacturers and designers have begun to propose vehicle–user communication solutions, motivated by the anticipated arrival of autonomous vehicles (AV).

Therefore, HMI solutions have been lately explored to increase safety in the interaction among vehicles and VRUs. The main focus is on allowing cars and pedestrians to communicate effectively, thus avoiding uncertainty in intention estimation. External human–machine interfaces (eHMIs) arise as a solution for the vehicle to transmit information to potentially dangerous agents in the vicinity. 

Similar to non-external interfaces (HMI), eHMIs must display information of interest, comprehensible and at the time of need, in addition to performing message category management. Road safety must be the objective of the introduction of this equipment on autonomous vehicles. 

Although eHMIs have the potential to benefit road safety even in the current human–driver paradigm, they will become critical with the emergence of AVs, when the current channels of communication between drivers and VRUs (e.g., gestures) are no longer feasible [9]. Smooth integration of AVs in society will require them to be able to make their decisions clear to all the participants involved in the traffic situation.

In some respects, the problem can be seen as a particular instance of human–robot interaction (HRI), an issue that has been extensively studied in the literature [10]. The development of the robotics industry has demonstrated the possibilities that exist when using intelligent devices, which provide solutions that simplify deployment and enable options to test various methods of human–vehicle interaction [11]. However, eHMIs have some distinctive features that make them unique, such as the short time frame available to establish the communication and the variability and lack of structure of traffic environments.

In this paper, we will focus on the design of effective eHMIs in the context of automated driving with the aim of proposing some guidelines due to the lack of standardization. Our contribution is threefold: First, we will explore the rules governing the communication between drivers and VRUs (especially pedestrians) and the decision-making process involved in their interaction, focusing on the problems that will arise with autonomous vehicles. Second, a review of reference literature papers will be conducted to get a complete picture of the characteristics of the different types of HMI. Finally, based on the conclusions drawn from the previous analyses, we will propose clear guidelines for selecting and designing the most appropriate eHMI for each application. 

The paper is organized into six sections, where Section 1 is dedicated to introducing the paper. Section 2 provides an overview of the current status of driving automation. Section 3 describes the vehicle–pedestrian interaction and the factors involved in the decision-making of both kinds of agents. Section 4 reviews the eHMI technologies most frequently used in industry and literature. Section 5 carries out a complete review of the work done to date about the effectiveness of different eHMI alternatives in experimental environments. Section 6 provides concise guidelines to design a proper eHMI due to the absence of ISO rules. Finally, Section 7 summarizes the conclusions and open lines of research on this issue.

## 2. Automated Driving 

The automotive industry is growing bigger and preparing for the new era of autonomy. AVs are expected to reduce the number of accidents generated by human errors [12], mainly due to fatigue, stress, or other factors such as drinking and driving. Because of the benefits that this step forward promises, the entire industry is in tune to take this step in the short-to-medium term. Technological giants such as Apple or Google [13], or parts suppliers such as Aptiv [14] or Bosch [15], besides new car brands such as Tesla [16], are leading the way in providing elements for the consolidation of autonomous driving. Ultimately, its success will depend on two types of factors: technical and human.

### 2.1. Technical Evolution 

Although the mere advance of technology will not suffice for transformation towards autonomous driving, the availability of robust automation systems is one of the most critical requisites. 

The first DARPA challenges [17], held at the dawn of the vehicle automation technique, are far away, and today more and more car manufacturers are providing automated solutions that range from parking to motorway navigation. However, there is still a manifold of challenges to solve.

The Society of Automotive Engineers (SAE) proposes different levels of autonomous driving based on their functions or degree of interference of the automaton on autonomous driving [18]. The first three levels require monitoring by the driver. In contrast, the next three are truly autonomous, graduated based on the temporality of the autonomous task: punctual, in the majority of situations, and completely autonomous in all situations, respectively. Table 1 summarizes the properties of each of the six levels. 

Due to the diversity of situations that can be found in driving environments, reaching the higher levels, where the vehicle must perceive the environment and act accordingly by itself, is a challenge that will have to be solved in the years to come. For our study, we will consider that a self-driving system is one that is endowed with an automation level of 3 or higher. A level of conditional automation allows for secondary activities such as sending text messages and making phone calls.

In any case, technological development must be accompanied by social acceptance of the product. Widespread use will also depend on the perception of non-buyers of the technology: pedestrians, passengers, and other road users.

### 2.2. User Acceptance 

Even after the technology that allows the complete automation of the vehicles is developed, some human-related concerns will have to be addressed; especially, user acceptance, in which vehicle–user interaction is expected to play a fundamental role. It is noteworthy that most of the population has different roles in different moments of interaction on the public road; for instance, drivers are also pedestrians when they walk on the street.

Several studies have been carried out in the last five years to find out the general population’s perception of autonomous driving. These studies analyze public opinion and perception of AVs through surveys in focus groups. Although they do not show a clear pattern when assessing age and gender differences, some of them reveal an apparent distrust of autonomous vehicles [19].

A recent survey of individual preferences for vehicle automation [20] reports that acceptance of vehicle automation decreases as automation capabilities increase. The study, with over 600 US drivers, shows that a large percentage would accept partially autonomous driving, but only 15.5% would be satisfied with obtaining fully autonomous driving. Nevertheless, it also suggests that the general acceptance of technological changes in vehicles increases as these systems are developed and implemented, and autonomous driving has only started to accelerate in recent years. It has been shown [21] that most comments about the topic made by readers on US and German media portals still focus on the general characteristics of autonomous driving. There is, however, a growing interest in analyzing specific user scenarios and promoting the use of autonomous driving in the context of improving people’s mobility.

A more recent survey project [22] shows greater acceptance and interest in this type of vehicle. In a survey of pedestrians with actual experience, only 6% of the group felt that autonomous vehicles could not improve road safety, and 67% said they thought it could be a means of reducing traffic accidents. 

Although autonomous vehicles are mainly perceived in a positive way, there are also responses of skepticism and distrust towards the possible development of autonomous driving in the real environment, mainly associated with negative consequences due to danger or even the possible loss of freedom. However, this stance of rejection is typical among the attitudes towards technology, as is reflected in the results of other technology acceptance studies [23].

Regarding VRUs, it is argued in [22] that the degree of driving aggressiveness of AVs impacts how pedestrians perceive them in crossings. In marked pedestrian crossings, AV driving behavior has little impact on confidence, but in unmarked pedestrian crossings, AV driving behavior is a major determinant of confidence.

Despite the significant value of the insight that these works provide into user acceptance, they face a common challenge: currently, the general public does not have access to AVs, which provides inaccurate information to the perception of respondents. It is not trivial to convey to respondents the precise uptake or understanding of the use and consequences of using these vehicles. At best, surveys are conducted in Virtual Reality (VR) environments with steep learning and understanding curves. In other cases, studies employ photographs or videos, where the respondent has to do a retrospective exercise to an unknown place. In both cases, they require an exercise in imagination towards a future in which autonomous vehicles populate the roads.

## 3. Vehicle–Pedestrian Interaction and Decision-Making 

Communication between road users is an essential factor in traffic environments. Usually, non-verbal cues are used to exchange information quickly and unambiguously. This communication strategy becomes infeasible when dealing with AVs, giving rise to a humanistic problem that must be resolved before the launch of technical advances. We refer to the pedestrian–(autonomous) vehicle interaction. The most problematic scenario will be that of unmarked roads, especially urban ones, especially considering the disagreement with the rules that some pedestrians may exhibit. 

Therefore, understanding the cues currently used for driver–pedestrian communication and the motivation behind the decisions made by both parts (i.e., braking or accelerating, stopping, or continuing walking) are prerequisites in the design of effective eHMIs. There are different profiles and different rules in the pedestrian environment. The first social study of driver–pedestrian interactions is over 40 years old [24], and it claims that there are two kinds of traffic rules: formal and informal. Both kinds of rules will be described below.

### 3.1. Formal Rules

The traffic regulations governing the road environment, which motor vehicle drivers must accept and follow, also define the interaction with other users. This interaction has been studied and, in works such as [25], the differences by nationality are set out. Differences between countries in yielding regulation pose a significant challenge in the application of standards for interaction with low-level autonomous driving systems. In Germany, when the right-of-way has priority for pedestrians, there is a very high willingness on the part of drivers to yield to pedestrians. In China, the preference of pedestrians is to wait until the vehicle stops due to mistrust. This diversity complicates the design of rules for interaction with autonomous driving as a world standard.

### 3.2. Informal Rules

The other area of rules is informal, used in the absence of specific regulations or to require more information from the interaction. Pedestrians use visual contact with the driver for a final decision to cross in, in most cases, even if there is a preferred (western) pedestrian crossing.

Although they are not essential with traffic regulation legislation, several studies confirm that non-verbal communication cues are present in most pedestrian–driver interactions [26,27,28]; for example, the search for visual contact with the driver by the pedestrian to make sure of the cession. The driver also produces gestural signals to favor the decision to cross or to resolve situations that may appear ambiguous.

Typically, the development of new technologies aims to solve or improve efficiency in the face of problems or tasks. However, these changes may lead to the emergence of new problems as, in this case, the absence of a driver who can perceive and provide relevant information in certain situations such as passing [12]. Thus, the necessity of a new form of interaction arises. A straightforward solution would be to use sensors to identify the indications made by the pedestrians and then react to them appropriately, including the possibility to respond if necessary. However, gestures by pedestrians asking for more information are usually too subtle and ambiguous to be detected with the current sensor technology [28]. Instead, the current focus of development is on sending information through external interfaces without prior information requests. Of course, advances in sensors and artificial intelligence [29] suggest that, in the medium-long term, we will be able to obtain mixed solutions with eHMIs that inform the pedestrian but at the same time detect these ambiguous pedestrian gestures.

## 4. eHMI Technologies

Foreseeing a future filled with autonomous vehicles, automotive companies and researchers have considered and developed new ways for intent communication between pedestrians and vehicles based on external interfaces. The following lines address some of the most relevant proposals.

eHMIs will be very useful in certain circumstances in which pedestrians or external users require extra information. As stated, pedestrian behavior is less predictable due to the nature of their movement, lack of training, and the ease, in certain circumstances, of not following the rules. eHMIs can help reduce uncertainty in their behavior.

Moreover, it has been observed that there are common factors that determine the feeling of security of pedestrians in traffic situations, such as vehicle kinematics and sizing. For instance, a recent experiment using augmented reality in the Unity environment [30] tested three different vehicle sizes (small, medium, and big), concluding that the safety perception in front of a car decreases as the size of the car increases by a small value; in previous works not focused on autonomous vehicles [31], the same results are found. According to [22], this feeling of insecurity in the tests performed increases when the autonomous vehicle was programmed not to yield.

Improving vehicles’ communication capabilities towards external users is the only way to make pedestrians feel safe and, therefore, accelerate autonomous vehicles’ acceptance. To this end, different technologies are being studied and will be introduced below.

### 4.1. Technologies

Leading manufacturers have made an absolute commitment and understood the need to develop eHMIs together with autonomous vehicles. Research stakeholders have produced diverse eHMI design concepts that vary in both their implementation and their position, color, and technology, even in the tone of the message.

In the automotive domain, the visual channel remains the primary way to communicate information to the driver. With the widespread adoption of AVs, this information modality will still be easily perceived and correctly interpreted by passengers and external users.

#### 4.1.1. Display

**Figure 1:**
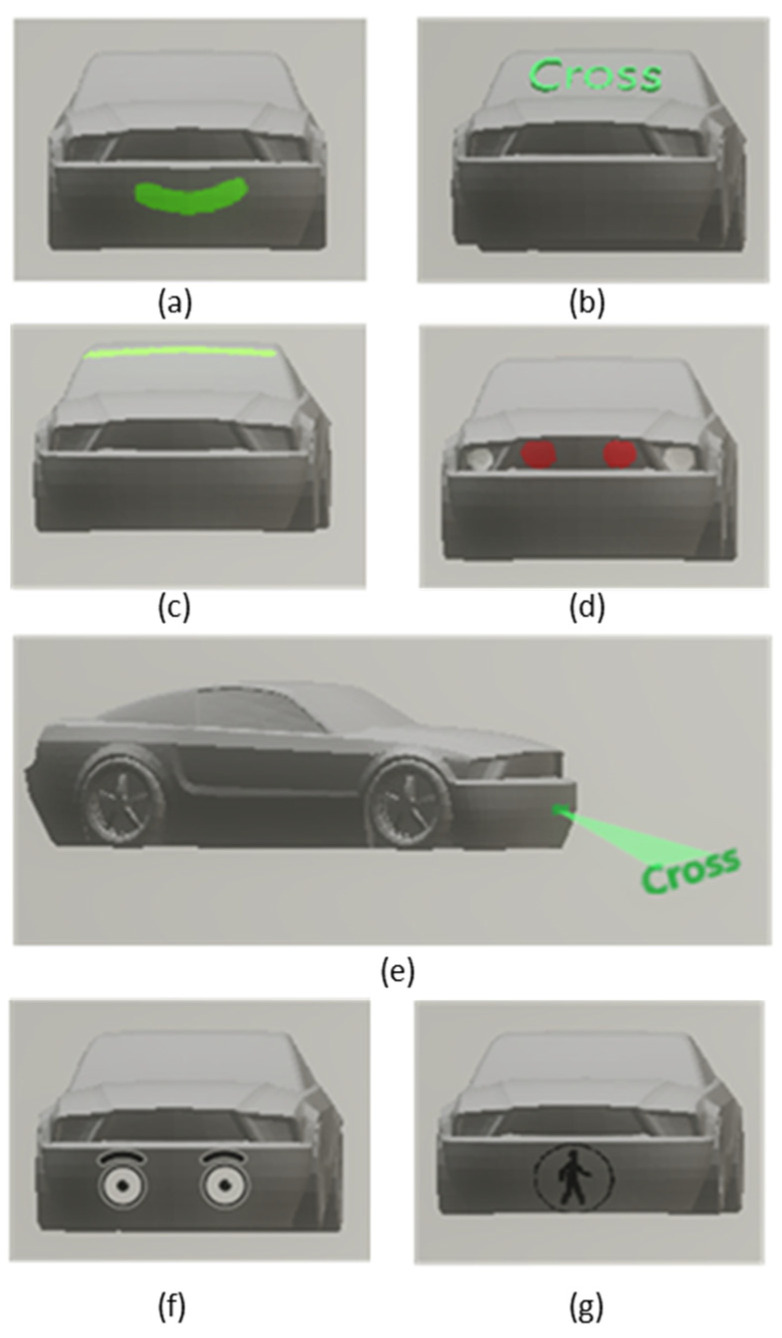
Different eHMI technologies: (**a**) smiling display: using a smile on the front of the car to deliver information to the external user, (**b**) display on the windshield showing in text information what the pedestrian should do, (**c**) LED light strip on the windshield in different motion sequences according to the message, (**d**) front brake lights: send brake information to the front, so you do not rely solely on the perception of deceleration, (**e**) projection of messages on the road: with text elements or crosswalk, enabling the “safe crossing” option to the pedestrian, (**f**) vehicle has a human-like appearance with eyes to show detection, (**g**) icon of yield on, as well as pedestrian crossings.

In 2012, Google obtained a patent on the communication systems of autonomous vehicles with the rest of the users in the environment, mainly by showing icons or text messages, although they also added audio options [32]. In the automotive industry, as mentioned above, several brands are researching these interfaces. However, the highlight in display technology is Nissan with its so-called IDS (Intelligent Driving System) concept [33], which illuminates messages on the windscreen. The Swedish company Semcom proposed a very friendly and somewhat humanized concept, showing a smile when vehicle yields [34]. This smile appears on the front grille of the vehicle, confirming detection and that the vehicle will stop, as illustrated in Figure 1a.

In addition to serving as a passive warning signal, the possibility of adding dynamic information using the same screen technology is also being studied, improving efficiency and understanding between the pedestrian and vehicle. Reference [35] presents two eHMI concepts: a vehicle-mounted screen with an icon informing about the possibility of passing (walking/crossing or not walking, as shown in Figure 1b) and another screen (also mounted on the vehicle) showing the speed of the vehicle. It is argued that if pedestrians are made aware of the status of the vehicle, e.g., showing a continuous detection warning or the approaching speed, decision-making is benefited, and the perception of safety increases.

#### 4.1.2. LED Light Strip

Instead of displays, ref. [36] presents a multi-modal eHMI made of two elements: a LED light strip, similar to the one shown in Figure 1c, and more speakers placed on the car, in the position of the windscreen and hood. Pedestrian detection is transmitted by means of flashing lights, and the message and intention use fixed lights, with colors as red (“not stopping”), green (“stopping”), and yellow (“starting”) lights; a voiceover is also added.

In [37], where a more basic interface was studied, good results were obtained using a vertical LED light strip. In this case, the vehicle had the appearance of an AV, and a satisfactory evaluation was obtained in terms of the perception of safety and comfort when extra information was received. Similarly, ref. [38] evaluated the “Autonomous Vehicle Interaction Principle” (AVIP), again proposed to use an interface to transmit information on whether the standalone mode is active and the intention of the vehicle, providing a safer pedestrian user experience. The design consists of a strip of RGB LED light (red, green, blue) located at the top of the windscreen, where three clearly differentiated messages are transmitted by means of color combinations (white/yellow) and light movement.

Both works share the position and use of solid colors that do not require training, such as red or green, allowing all road users to understand when it is safe to cross the road without training. Ford [39] decided to investigate without colors using blinking modes, full solid light, or moving lights that run along the LED strip similar to the famous TV show Knight Rider. This well-known light animation has been investigated in other works [40], although it was eventually proved a suboptimal option when the whole age spectrum is considered, compared with alternatives such as front brake lights, smiley, or text. 

#### 4.1.3. Front Brakes Light

The front brake lights concept, depicted in Figure 1d, seems intuitive and relatively easy to implement: adding bulbs in the front reflecting the same information as the rear bulbs. Some patents were already published in the 1920s when even the rear brake lights were still a novel technology. Later, a patent from 1998 [41] brought back this technology for communication with pedestrians or cyclists. 

Front brake lights are a concept that has been proposed before in different forms and formats, being considered as an innovative system in [42]. A complete study was published in 2018 [43] where it was shown that front brake lights helped participants identify the speed and deceleration of an approaching vehicle, speeding up the decision processes concerning pedestrian crossing decisions.

#### 4.1.4. Projections

The projection technology has already been implemented for in-car HMI, communicating to the driver. While most eHMIs consist of lights or a screen implemented in the front of the vehicle, as seen in the previous sections, relevant information can also be projected forward, as illustrated in Figure 1e. Thus, the Mercedes-Benz F015 concept [44] uses a laser system to project relevant information onto the road; for instance, a virtual pedestrian crossing is created whenever a person waiting to cross is detected.

Instead of making a hologram or reflection of light on an object or the road, the AutonoMI project [45] proposed reflecting on the individual by tracking him as he crossed, showing that he had been identified.

#### 4.1.5. Visual Contact Simulation

Pedestrian tracking, simulating the eyes’ movement of the false driver, has been one of the interfaces launched in a novel way, as shown in Figure 1f. Initial approaches consisted of following the pedestrian, when detected, using lights; however, other works have gone further and have included realistic eyes on the car, as exemplified in Chang et al. (2017) [46], evaluated this tracking interface, simulating human eyes to replace the lack of visual contact between driver and pedestrians. The idea is to use eye contact (machine–pedestrian interface): when there is an intention to yield, the headlights turn and look at the pedestrian to inform the pedestrian of their detection and communicate the vehicle’s intention; otherwise, the eyes look ahead following the road. The resulting surveys show a good acceptance of this interface, as well as an increase in safety.

#### 4.1.6. Audible Interface

Most eHMI technologies use visual interfaces because they are the maximum exponent of signaling in car traffic; additionally, sound can be inaudible in rush hour. However, some surveys studied in the next chapter complement visual information with sound cues, extending the message transmission to the visually impaired. Mental models, such as the ones used in audible traffic lights, are used; for instance, Costa et al. [47] experimented with variations in the tempo of the sound. Other concepts also include clear and concise messages such as “cross” or “wait.”. Deb et al. [48] included the vehicle horn, music, and a verbal message with a less imperative message: “safe to cross.”

### 4.2. Visibility

Visual technologies will require a detailed study of their visibility. Most studies focus on the type of technology or symbolism; however [49], experiments with visibility by experimenting with the optimal position of the screen or eHMI in all conditions that can occur in real traffic to ensure sufficient information for pedestrians and increase their perception of safety. The study is based on an initial analysis with a 360° camera and a later 3D simulation with tangible measures. More than 332 simulations were performed to observe the most visible areas in the car for the pedestrians under different parameters, such as different directions of the pedestrian crossing (i.e., left to right or vice versa). It was concluded that, when a pedestrian crosses the street, the perception of the front and side parts is approximately balanced (50%/50%) for the first car but leans towards the side in the second (33%/66%), third (15%/85%) and farther vehicles. This conclusion does not affect the relevance of frontal eHMIs but proves that they are not enough in urban environments.

On the other hand, while most studies focus on pedestrians as the largest group of VRUs, AV-cyclist interactions present specific challenges that cannot be overlooked and are frequently related to visibility concerns. As mentioned in [25], when pedestrians are involved, movements can be assumed slow and, thus, continuous visual contact is feasible. However, these restrictions do not apply to communication between AVs and cyclists, especially when both parties are moving quickly in the same direction; in those cases, vehicles are often behind the cyclist’s line of sight, making it impossible to rely on visual cues solely. 

## 5. Effectiveness Assessment

There is a vast literature on human–machine communication, but we will focus on VRU-AV communication. Different investigations have been carried out to determine pedestrians’ reactions to different eHMIs: Who better than pedestrians can help us in their design?

The testing possibilities are limited, but still, different environments can be tested through surveys. In recent years, studies have focused on virtual reality and even some real traffic environments.

### 5.1. Image or Video-Based Surveys

The first approach to evaluating a future scenario could be to show the participants multiple images depicting prototypes and ask their opinion and degree of acceptance of the different options. For instance, the viability of crossing is studied in [50], where a methodology based on Amazon Mechanical Turk (AMT) is employed. AMT is an integrated web-based task presentation platform and a participant compensation system. It provides access to a large group of potential participants at a modest cost per participant. Numerous studies on this platform [51] show good performance, especially in psychology and other social science research, as participants are diverse and more representative of a non-university population than traditional samples. The stimuli (i.e., the images to be surveyed) were created by superimposing 30 different designs on the base image, with each design being animated. The aim in [51] is to validate the AMT-based proposal to analyze pedestrian–vehicle interfaces, being cost-effective to identify design concepts that may be appropriate for further development and testing.

Similarly, MTurk was used in [52] to study the usability and preferences in color and animation in traffic negotiation situations. Four hundred participants helped to understand the comprehensibility of a light-band eHMI with a combination of five colors and three animation patterns for a standalone car. The results suggest that while red and green have immediate associations with “stop” and “go,” the meaning of these messages is not always exact and can lead to confusion. Cyan blue, on the other hand, is a neutral color and, therefore, may be more appropriate. Animations were found to have less impact than colors, although uniform or flickering animations are generally more favorable than laterally sweeping ones. The result of this work highlights the importance of colors and animations in standardization.

The use of two screens placed on the front of the vehicle is investigated in the work of Song et al. [53]. The participants saw videos of the autonomous vehicle in real traffic, where one of the monitors was showing icons, such as the zebra crossing sign, and the other was used to transmit either an informative message (“Ok”) or an order (“Go”). The results did not reveal a significant difference between both alternatives, although more frequent crossing was observed when the vehicle was endowed with any of the alternative eHMIs.

A comprehensive two-purpose survey was conducted in [54]. In the first part of the survey, a study was based on showing 28 images through drawings and video of existing patents, concepts presented by the automotive industry. It turned out that textual eHMIs were generally considered to be more efficient, not requiring any learning. On the other hand, non-textual ones show a clear acceptance of projection, while those placed on the car with icons or movements need a previous explanation; even some light can be confused with an ordinary sensor. The second part focuses on the effects of text perspective, comparing the egocentric point of view of the pedestrian (“Walk,” “Don’t walk”) with an allocentric perspective (“It will stop,” “It will not stop”), together with different colors. Respondents were asked whether they felt safe to cross in front of the AV. As in the first survey, the text eHMIs were more persuasive; in particular, the text “Walk,” showing an order of action, was the most valued in safety. The conclusion is that the textual eHMIs are considered the clearest, which poses a dilemma because textual instructions must be accompanied by linguistic responsibility, readability, and technical feasibility.

A different study combining video simulation involving 26 participants and discussion with six naive pedestrians to establish the evaluation criteria was presented in [55]. In the first phase, the evaluation criteria were established: recognizable, unambiguous, interaction, comfort, and intuitive understanding. In the second phase, with a larger group, the different HMI designs were evaluated by creating variations in position, type, message coding, and technology. It is demonstrated (again) that self-centered instructions to the pedestrian are preferable to vehicle status information. Regardless of message coding as text or symbol, the lowest number of incorrect responses (31%) was for projections,34% with screen use (34%), compared to the significant difference of 78% for LED strips. However, the need for future work to focus on scenarios with multiple autonomous vehicles reflected on the road is highlighted, which could generate insecurity.

Further studies confirm that the information obtained by the eHMI is interesting for safety; for example, in [56], different levels of eHMI are considered in terms of information: reporting activation of the autonomous mode, showing its intentionality (stop or go), and perceiving the pedestrian (simulation of eye-tracking). Sixty-two pedestrians responded that any eHMI contributes to a more positive feeling, the state being obvious information, but causing a sense of intelligence to the car when it has an eHMI with intentionality.

Chang et al. [57] did not focus on innovation but rather on comparing technologies that had already been developed. By carrying out a survey based on animated videos, five different eHMI modalities were compared in terms of their effectiveness in communicating the car’s intention. The first of the studied alternatives was, surprisingly, inspired by human appearance: the headlights showed eyes when detecting a pedestrian and followed him during the crossing. A smile was also used, showing in the grill when the intention was to give way. The third made use of a text message, in orange, showing “you may cross.” The fifth was more abstract, using a green flashing LED strip for yielding. Finally, a projection on the road was used to show the pedestrian a zebra crossing for guiding the crossing. The information based on textual language and the projection were the best evaluated in terms of ease of understanding the message.

Charisi and others [58] evaluated various interfaces in relation to their potential use for children. The reasoning is twofold: first, they are part of the traffic environment, and second, if an eHMI is designed to be understood by a child, it is likely to be valid for any pedestrian. They used icons known as traffic light interfaces, traffic signs, projected crosswalks, children’s drawings with “Pass” or “Stop” signs, the use of icons known as pedestrians crossing, LED light strips, or human-looking headlights. An image questionnaire was administered to the participating children, who were assigned the task of reporting on their perceived right-of-way based on the design shown. Designs based on known order systems, traffic lights, standard road signs were preferred. 

Li et al. [59] focused on increasing the sample of urgency in risk situations with vulnerable road users. Displays were placed on the windscreen and radiator, showing three types of messages with two different interfaces: “safe to cross” (green or white fixed), “safe but not recommended” (amber or red flashing), and “dangerous to cross” (red), the first options being the same as an ordinary traffic light. The study was based on surveys conducted after watching animated videos showing vehicles traveling at relatively high speeds in urban environments (50 km/h). They were asked not only to comment on the design but also the urgency of the design. While the message of urgency for the most dangerous states was well received, the pedestrians admitted that the decisions were actually based on the kinematics of the vehicle rather than the use of the interface. These findings confirm that, as stated before, individuals have a high preference for making choices based on known elements, such as vehicle kinematics. 

The work of Zhang et al. [60] is notable for the position of the displaying elements, which were RGB LED strips aimed to show the intention of the vehicle. They were installed covering the front doors and the hood of the vehicle to tackle the possible lack of visibility in traffic. Videos were shown to the participants with the different combinations of the interface, based on colors and movement in the LED strip. They had to guess the message the vehicle was sending and the possible efficiency or usefulness in real traffic. They found that participants were able to infer that messages were intended to express the vehicle’s intentionality, but when five different intentions were presented, confusion appeared in intermediate states between stopping and moving forward. The solution that was finally deemed optimal made use of lines with movement in the direction of the road for acceleration or forward movement and in the opposite direction for vehicle deceleration.

Surveys carried out with simulated stimuli and from the tranquility of an external environment are a reasonable first step for the concepts. However, we argue that the validation and standardization of an interface of these characteristics should be carried out with concrete evidence using stimuli closer to a real environment. However, these good results contribute to a process of relatively fast acceptance with respect to the possible learning curve due to the great technological change.

### 5.2. Virtual Environment 

The WEpods were automated shuttle buses that were deployed in a campus environment in the Netherlands. The project enabled different experiments focusing on the vehicle–pedestrian interaction to understand reactions in a controlled environment, both based on pedestrian surveys [61] and virtual reality [62]. In both cases, pedestrians reported that they felt safe in the presence of WEpods. However, the action of crossing the street in an unmarked area with an automated vehicle caused distrust, and they preferred to use a pedestrian crossing.

Among the experiments carried out in virtual environments, ref. [30,63] stand out for being affordable using the same factors and the experiment reliability method based on the work by Witmer [64]. They also share the same visualization scenario, consisting of locating the subject on a curb in front of a pedestrian crossing two steps away, where participants could cross when they felt safe enough to do so.

In addition to the analysis already commented on the perception of the pedestrian in the decision to cross in terms of the size of the vehicle, ref. [30] also investigated the effect of external interfaces in the virtual environment through a head-mounted display (HMD) or virtual reality glasses. Twenty-eight participants stood on a virtual curb and observed a square of autonomous vehicles, with or without eHMI. The presence of an eHMI indicating whether the AV would stop or not significantly increased the security perceived by the interviewees compared to a situation where there was no eHMI. The text-based eHMI was found to be the best rated in general because other types of eHMIs required additional learning (e.g., smiley, front brake lights, Knight rider LED strip, etc.).

In [63], the 24 participants were immersed in a virtual environment, similar to that used in De Clercq et al. (i.e., an urban traffic scene) but with the disruption of wearing a motion-tracking suit. The movement tracking suit allows research into pedestrian behaviors related to body attention and hesitation. The study was carried out with a random behavior of the vehicles (yielding, not yielding) and the type of eHMI (None, Textual, Front Brake Lights). Participants were asked to cross only when they felt really safe to do so. Thanks to the suit, the quantification of the results is more accurate, showing that the forward speed results (from the pelvis) were on average higher with the existence of an eHMI in the vehicle assignment. The importance of distance is noted, as crossing in a space of 20 m between cars was dangerous without the indication of an eHMI of the vehicle detection. However, crossing at a distance of 30 m between cars without an eHMI was possible. Most participants started crossing directly as soon as they had the opportunity, regardless of whether the car had an eHMI or not. These findings indicate that motion detection with the suit allows for pattern modeling that is not evident in sensorless reporting surveys.

The work of Otherson et al. [65] focuses on two parameters: the coding of messages as abstract or iconic and the animation of visual signals. The installation of the interface was proposed on the radiator grill, presenting four different designs. The information provided by the elements was based on the detection of the pedestrian (activated at 50 m) and the intention of letting the vehicle pass by, making designs with static and dynamic elements. Better results were obtained with the latter, making the animated ones more understandable and informative against the static ones, which required a not very intuitive interpretation. It highlights the effective use of human appearance, using the eye as a dynamic interface in the detection of the pedestrian.

Deb and others [48] introduced two technologies, visual and audible. The visual elements were similar to the works already mentioned. However, they did an interesting exercise with the audible part: they tried to make car sounds like the horn, music and sending messages through voice messages, ensuring the possibilities of crossing, as it was expected the voice messages were more popular, understanding that the horn could have a psychological connotation of punishment. This was reflected in the measured crossing times, where they were shorter for the music or voice message technologies and longer when the horn was used. 

Mahadevan and others [36] experimented in a virtual environment and with participants selected according to various criteria in order to have a rich spectrum of different types of pedestrians, such as different age ranges or experiences. In addition to the visual mode and the use of sound, mobile technology and tests with elements in the infrastructure were added. The study led to a better understanding of the virtually endless possibilities that can be obtained by the good use of elements on the network, although such a system would also limit the crossing to the defined areas. In a world where almost every pedestrian has a mobile phone, the use of these devices as an interface was also considered. However, the option of using a mobile phone was the most inefficient as reported by pedestrians, and the best evaluated was the other novel alternative proposed, i.e., the use of elements installed on the road. 

It has been observed that, paradoxically, the development of this technology may increase the gap with people with disabilities if not dealt with properly. As a matter of fact, works addressing the use of audio technologies for pedestrian–vehicle interaction are very limited. However, some works such as [58] give some initial ideas; for example, it states that too long audio messages were not generally accepted. However, a recent study presented in [66], which focuses on people with vision impairments (VIP), provides a different point of view. In this study, based on a virtual environment and a movement measurement suit, it is shown that the use of extended audio messages specifying intent and instruction, such as “I’m standing and you can cross over,” are best rated by VIP. Nevertheless, it is clear that more research effort still needs to be devoted to this issue before deploying eHMI technologies. 

Finally, some studies have focused on the particularities of the interaction with cyclists, as is the case with [24], where different vehicle–cyclist interfaces are tested in a virtual environment. The proposed technologies are similar to those applied in the communication con pedestrians, including laser projections on the road and the use of the windshield of the vehicle as a screen to show that the cyclist has been perceived and its path is safe for circulation. 

In summary, studies using augmented or virtual reality aim to combine the best of both worlds, i.e., strict experimental control and highly realistic conditions. However, there is still a non-negligible gap with reality in situations that require quick decision-making in front of a self-driving car. Fortunately, in the last few years, we have begun to see studies carried out in real environments, as discussed in the following section.

### 5.3. Real-World Experiments

To get proper feedback on this new reality, an exercise called ghost driver has been proposed [67], where the human driver is engaged in other activities other than driving and, similarly to AVs, cannot give effective communication cues. To that end, they carried out a Wizard-of-Oz typology study to investigate the reaction of pedestrians by making them believe they were autonomous vehicles, introducing an invisible driver in black suits and modifications to the vehicle seat. On the other hand, a study on the non-need for eHMI was carried out in [68], which evaluated the intention to cross in a real environment using an autonomous vehicle (fake LiDAR) and yielded surprisingly good results: 100% of the pedestrians crossed without hesitation. There were no sensors, so the output could not be measured, and conclusions were obtained after reviewing the recordings. It is also worth mentioning that the tests were made after a roundabout, exhibiting the need for further research on these environments.

Lagström and Lundgren [69] did a preliminary study for the design of their eHMI, presenting the participants with different photographs and asking for their emotional reactions in an imagination exercise where they were walking through the city center and about to cross an unmarked pedestrian walkway after a car had stopped. It was observed that a “driver” engaged in activities other than driving or distracted, as was the case in most pictures, was used as an indicator that the vehicle was not about to move, enhancing the possibility of crossing by pedestrians. This misinterpretation of an automated vehicle could lead to an incident when dealing with an AV, thus motivating the need to install some indication of whether a vehicle is in autonomous mode and of its intentions. According to this, a simple eHMI based on an LED strip at the top of the windshield was designed and tested in real environments without traffic. Answers from participants showed that the system did not replace eye contact with the driver, but it was considered highly convenient to provide early-access information.

Clamann [35] performed tests in a real environment with traffic using a vehicle that the participants were led to believe was autonomous. An LCD was mounted displaying symbols for pedestrian crossing (Figure 1g), pedestrian non-crossing, off, and information, and experiments were performed at different distances in a pedestrian crossing and an unmarked crossing. An additional ANOVA test was carried out to compare with the results of the experiment in a real environment. Of the participants, 66% reported seeing the screen on the front of the vehicle during the experimental tests. However, only 12% reported that it influenced their decision to cross, consistent with the ANOVA results. Distance to the vehicle was reported to be more critical in the decision to cross, with 56% of participants indicating this as a factor. This result is consistent with previous findings that gap distance is the primary determinant of a pedestrian’s decision to cross.

For the work of Hensch et al. [70], drivers were also chosen without selecting a definitive spectrum, i.e., at random. The external interface was based on a LED matrix placed on the car roof, which only showed three types of message, based on the modification of the color and type of light of the matrix. The different modes selected were an information message about autonomous driving (cyan blue), a warning about the vehicle’s approach to the crossing area (flashing light), and the intention to yield with a solid light, moving in both directions, like the already mentioned Knight rider. The main result was a lack of understanding of the different modes and the need to learn, one of the points to avoid in this type of element.

Hudson et al. [71] and Costa [47] showed models displaying information based on a two-way LED display and a built-in speaker for audio information, sending voice messages. The visual part used both text messages and icons. The tests were carried out on an unmarked crossing area, and all the eHMIs were interesting compared with the option of not installing eHMIs. Costa [38] introduced the same text elements and visual icons, and, like Henscht et al., the pedestrians had been chosen at random. The speaker system emitted sounds based on the tempo standard of an ordinary traffic light. Again, in contrast to the non-existence of eHMI, positive results were obtained, with the messages offered by the vehicle as when asking not to cross.

In [72], experiments are conducted in a real controlled environment using a framework where posture detection is performed to discern the intentionality of crossing by the pedestrian when approaching a real autonomous vehicle. After detecting posture, an eHMI was used to inform whether the pedestrian was detected, with a symbolism common to current traffic lights (pedestrian crossing or not crossing). 

A summary of the eHMI-related studies discussed in this section is presented in Table 2, showing the eHMI technologies and message coding used in each of them.

## 6. Guidelines

After studying the multitude of interfaces designed in the context of pedestrian–vehicle interaction and realizing the importance of applying them in the design of solutions for autonomous vehicles [74], we aim to find guidelines for their design. The recent standard DIN EN ISO 9241-110:2020 [75] is not applicable as devoted to HMIs, without active interaction with the pedestrian. Moreover, eHMIs must be pertinent for a potential multitude of users. The general guidelines that we introduce here aim to provide some conclusion to the literature reviewed and discussed within this paper, with the sole purpose of facilitating summarized information for practitioners.

To the best of our knowledge, the optimal characteristics of an eHMI have not been established unequivocally before. Some even rely on communication through social robotics, such as humanoid traits that simulate the figure of the driver in manual driving.

In recent work [18], a series of 20 guidelines focusing on HMIs for driverless cars, many of them common to the current ISO rules regarding vehicles, were drawn. Based on this, we will provide in this section a list of ideas, must-dos, and assumptions for developing and implementing eHMIs. 

In summary, the requirements for an appropriate eHMI are listed below:

- Modes

Our first recommendation refers to the constant display of relevant information for the pedestrian. By continuously presenting some messages on the interface, the pedestrian would be continuously informed of the state and perception of the vehicle, as proposed in [76]. 

Changes in the status of the system should also be effectively communicated. In the different experiments reported in the previous sections, the detection of pedestrians is assumed to be perfect, as they focus on the humanistic issue. However, changes in the state of the system, such as those produced by uncertain detections, should be projected in a remarkable way.

Time is critical in this type of interaction, which should not require continuous attention from the message recipients. Pedestrians must receive coherent information and should not need to divert all their attention to grasp the intention of the autonomous vehicle, e.g., in street-crossing situations, but stay aware of other possible risks in the surroundings. This rule becomes even more important where the interaction occurs outside a designated area (i.e., outside a pedestrian crossing) [63].

The self-awareness system mode should be present. In the event of a problem with the sensors, just as the driver is informed, a warning message should be displayed, showing possible non-interaction with pedestrians.

- Position, readability, and typology

Position and visibility are crucial to bringing information to the pedestrians promptly and clearly. In general, they must adapt to the multitude of changes in the environment due to traffic, as well as changes in speed or distance as in [43]. The root of the problem is finding a common interface for all pedestrians since, unlike drivers, they cannot be assumed to have any training. From this point on, the eHMI system must be highly visible to the target to be informed, i.e., the pedestrian.

In the case of symbols, they must be easily legible from the perspective of the pedestrian. Commonly accepted or standardized symbols must be used to communicate the automation mode. It is also possible to use text messages to reduce the time needed for understanding, although the content must be as short as possible without omitting any necessary information [40].

Texts are preferred in most works because of their easy interpretability. For this application, words must be easily legible, taking special care of characteristics such as the style or font. Moreover, unnecessary ornaments and decorations should be avoided to allow easy and fast reading. Regarding the content, it must be considered that not all pedestrians may understand the language of the message to be displayed; also, different reaction times might be necessary to understand it. Additional voice explanations could complement the use of non-standardized symbols such as [48] or [36].

- Colors and lighting

The SAE [77] and UNECE [78] working groups on lighting and signaling requirements for automated vehicles show some initial findings on the advantages and disadvantages of using specific color spectra for AV communication with road users [79]. Analyses of different dimensions of light perception, such as visibility, discrimination, and uniqueness, suggest that blue-green, turquoise, or cyan are the most appropriate colors of choice for the eHMI to communicate the intent of an AV in traffic. 

The importance of colors is also reflected in [52]. These guidelines for general HMIs suggest that not many colors should be used, and they should be coded consistently with the message being given. As a part of the message, they should follow standard conventions and stereotypes. However, in recent work [54], with respect to color options for light-based HMIs, it has been proposed that the use of colors for HMIs (in particular for autonomous vehicles) is recommended not to interfere with colors already implemented or reserved for other purposes in vehicles by the SAE J578 and UNECE Regulation R-65 specifications. Therefore, colors such as red, yellow (amber), selective yellow, green, restricted blue, blue, and white would be prohibited for these applications. Nevertheless, the literature confirms that the use of stereotyped or mind-modeled colors [58] improves the interoperability of the message. We propose using stereotyped colors in the road environment (red, green, or amber) only when they are accompanied by icons, or even better, with text that correctly identifies the message.

Luminance should be adjusted based on the vehicle’s environment, taking into account its location, contrast, background, and layout. eHMIs are even more sensitive to light than driver-designed HMIs and must operate 24 h a day in different luminosities, which is expected to require periodic self-calibration.

- Communication channels

The level of urgency should be transmitted through color or mode features. Nevertheless, pedestrians should be able to gather relevant, precise, and cataloged information straightforwardly from the contextual situation. 

Different communication channels, producing different system outputs, must be considered. Color blindness should also be factored in the design, using redundant coding and avoiding red/green and blue/yellow combinations. 

Regarding people with disabilities, some works have started to focus on these specific groups in recent years. As for the design guides, the work done in [66], although based on VIP with the role of passengers, shows a way of working based on participatory workshops in collaboration with disabled people to meet the needs of blind or low-vision users. On the other hand, in [80], the authors conclude that the results of the workshops should be taken with caution. Their results showed no relation to the experimental phase, which, according to them, could be due to factors not considered on-site. Reviewing the procedure of the workshop, the cause might be in the reduced number of participants in the workshop, who, moreover, share a common profile: experts in accessibility. It would be desirable to increase the number of consultants (VIP or people with some kind of disability) and have different profiles based on age or previous knowledge. People without previous knowledge of accessibility or related to mobility will give a real first impression when facing these systems.

To ensure the universality of eHMIs, it would be necessary to carry out studies to evaluate the acceptability and use of these interfaces by children, as introduced in [58]. To avoid the participation of juveniles in tests in real environments, we propose the use of virtual environments, in which the participants will be able to feel the experience from the perspective of a game. Questionnaires should be designed following the general recommendations in [81]: keeping questions as short as possible, reducing the number of response options, without ambiguity, and using vocabulary that is relevant to the age group. Questions should be very literal, avoiding mid-points on scales. The main idea is to create an environment where children can be themselves, and self-completion becomes fun.

Similarly, the more general case of guaranteeing the applicability of the systems to the whole range of pedestrian profiles, as well as people with disabilities, will require separate consideration. However, the concurrent use of different modalities, each targeting a different human sense, seems a promising approach. Table 3 summarizes the guidelines defined in this section.

## 7. Conclusions and Open Challenges

The introduction of autonomous cars on the road comes with one of the greatest challenges ever faced in the road environment: the interaction between them and other road users.

This work provides further insight into several aspects of the design of eHMIs as a response to this challenge, offering a broad collection of works demonstrating its potential to improve the interaction between pedestrians and autonomous vehicles. In general, it has been shown that eHMIs increase the efficiency of the activity of crossing the road, offering extra information. In addition, certain types of eHMI have been shown to affect pedestrian decisions when crossing the street, in the sense that pedestrians feel safe to cross when it is safe. For these reasons, eHMIs are one of the driving forces that must make possible the acceptance of AVs in the face of a major change that may occur in the coming years.

Moreover, we present some guidelines for the development of eHMIs that improve the current communication with the commitment to obtain safer and more efficient roads and populated environments.

However, our work has also revealed the existence of several pending challenges on which research should focus during the coming years. Hence, the distance available to cross the road with respect to the vehicle and the size of the vehicle still dominate the effect of the eHMI. The perception in the virtual environment is the same, producing a prevalence of decision making in this perception of the environment over eHMI, stating that eHMI presents extra information. This shows that we have not found the ideal eHMI; hence, more research is needed.

As shown by most works in the field, the main challenge is the need for comprehensibility of the interface, even avoiding the need for learning: eHMI should be unequivocal in understanding. As studied, the preference is text or projection on the road. These would present an extra guarantee of safety, especially if showing a clear message such as “walk” or “don’t cross.”

Based only on the latest, it seems that cyan blue is the color of choice. However, there is still a knowledge gap regarding the contextual effectiveness of the cyan color recommended in an eHMI to communicate with other road users. 

Future work in the field should also focus on the consensus of all pedestrian users. It should be achieved without introducing differences between pedestrians to the eHMI set; just as we use standard signs and bought by all drivers, pedestrians must have this standard. Most work focuses on young or middle-aged adults, but we must understand that pedestrian groups are formed by all age ranges that make up society, and groups such as children or the elderly must be assessed in surveys.

Finally, aligned with the promotion of inclusion, further investigation is needed in the design of multi-modal interface designs in which different sounds, and not just visuals, should be used to increase social inclusion.

## Figures and Tables

**Table 1:** Levels of driving automation Society of Automotive Engineers (SAE) International.

Level 0	Level 1	Level 2	Level 3	Level 4	Level 5
No automation	Driver assistance	Limited automation	Conditional automation	High automation	Full automation
The driver is the only controller all the time	The responsibility for driving is shared between the driving system and the driver. The driver must be available to take full control at any time	The vehicle can have occasional control of the vehicle in reference to the lanes and the speed. Monitoring by the driver is mandatory.	The vehicle has full control in certain limited situations and can inform the driver when to take control again.	The vehicle has full control over almost the entire journey, in most conditions. No operation by the driver is required.	The vehicle has total control without the need for any operation by the driver in all conditions.
These are driver support features			These are automated driving features		

**Table 2:** List of all surveys of all experiments shown in this paper sorted by the experiment type, environment, and message coding method in alphabetic order.

Study	Year	Stimulus	Type	Message Coding
Ackermann et al. [55]	2019	Survey, video or pictures	Display, LED light strip and hologram	Lights, textual and icon
Bazilinskyy et al. [54]	2019	Survey, video or pictures	Display, LED light strip, hologram and others	Textual, icon, sounds and others
Chang et al. [46]	2017	Survey, video or pictures	Rotating vehicle lights	Human appearance
Chang et al. [57]	2018	Survey, video or pictures	Display, LED light strip, hologram and rotating vehicle lights	Lights, textual, and icon
Charisi et al. [58]	2017	Survey, video or pictures	Display, vehicle lights and others	Lights, textual, and icon
Dey et al. [52]	2020	Survey, video or pictures	LED light strip	Lights, textual, icon
Faas et al. [56]	2020	Survey, video or pictures	Display, LED light strip and hologram	Light, icon
Fridman et al. [50]	2017	Survey, video or pictures	Display, LED light strip, hologram and others	Lights, textual and icon
Troel et al. [49]	2019	Survey, video or pictures	LED light strip	Lights position on the doors
Zhang et al. [60]	2017	Survey, video or pictures	LED light strip	Lights
Petzoldt et al. [43]	2018	Survey, video or pictures	LED light strip	Front brake Lights
Song et al. [53]	2018	Survey, video or pictures	Display	Textual an icon
Rodriguez et al. [61]	2017	Augmented reality	LED light strip	Lights
Böckle et al. [37]	2017	Augmented reality	LED light strip	Light
de Clercq et al. [30]	2019	Augmented reality	Display, vehicle lights and others	Lights, textual and icon
Colley et al. [73]	2020	Augmented reality	Speaker	Sounds for VIP
Deb et al. [48]	2018	Augmented reality	Display	Lights, textual, icon and sounds
Hedlund et al. [40]	2019	Augmented reality	LED light strip	Blinking modes
Hudson et al. [71]	2018	Augmented reality	Display	Lights, textual, icon and sounds
Kooijman et al. [63]	2019	Augmented reality	Display, vehicle lights	Textual and lights
Othersen et al. [65]	2018	Augmented reality	Display	human appearance
Alvarez et al. [72]	2020	Real environment	Display	Icon
Clamann et al. [35]	2017	Real environment	Display	Icon and text
Costa et al. [47]	2017	Real environment	Cardboard, speaker	textual, icon, sounds
Habibovic et al. [38]	2019	Real environment	LED light strip	Lights
Hensch et al. [70]	2019	Real environment	Display	Lights
Lagstrom et al. [69]	2015	Real environment	LED light strip	Blinking modes
Mahadevan et al. [36]	2018	Real environment	Display, LED light and others	Lights, textual, icon and human appearance

**Table 3:** Summary of the guidelines obtained from the literature review.

Requirements	Guidelines
Modes	Continuous display of status information [76]
	Notification of relevant status changes [63]
	Pedestrians should not divert all their attention to the AV [63]
Position, readability, and typology	Well-visible position for the pedestrian, even in different traffic situations [49]
	Use of symbols and icons that are easily interpretable, without the need for ornament [40]
	Texts as short as possible [40]
	Additional voice to help to deliver the message [48,36]
Colors and lighting	Use of eHMI-specific colors to help to identify these systems [79] and/or mind-modeled colors to help to deliver the message [58]
	Luminance must be considered to guarantee readability. Self-calibration may be necessary.
Communication channels	Prioritization of messages: urgency must be clearly shown
	Design targeted to the full range of pedestrian profiles [58]
	Inclusion of people with disabilities (i.e., visual impairments), e.g., by using different output modalities [80]

## Data Availability

Data sharing not applicable. No new data were created or analyzed in this study. Data sharing is not applicable to this article.

## References

[1] Bevan N., Carter J., Earthy J., Geis T., Harker S., Bevan N., Carter J., Earthy J., Geis T., Harker S. (2016). New ISO Standards for Usability, Usability Reports and Usability Measures. Human-Computer Interaction. Theory, Design, Development and Practice, Proceedings of the International Conference on Human-Computer Interactio, Toronto, ON, Canada, 17–22 July 2016.

[2] Mahmud S., Lin X., Kim J.-H. Interface for Human Machine Interaction for Assistant Devices: A Review. Proceedings of the 2020 10th Annual Computing and Communication Workshop and Conference (CCWC).

[3] Schmidt A., Dey A.K., Kun A.L., Spiessl W., Schmidt A., Dey A.K., Kun A.L., Spiessl W. Automotive User Interfaces. Proceedings of the CHI ’10 Extended Abstracts on Human Factors in Computing Systems.

[4] Alonso Raposo M., Ciuffo B., Dies P.A., Ardente F., Aurambout J.P., Baldini G., Baranzelli C., Blagoeva D., Bobba S., Braun R. (2019). The Future of Road Transport—Implications of Automated, Connected, Low-Carbon and Shared Mobility.

[5] Harper C.D., Hendrickson C.T., Mangones S., Samaras C. (2016). Estimating potential increases in travel with autonomous vehicles for the non-driving, elderly and people with travel-restrictive medical conditions. Transp. Res. Part C Emerg. Technol..

[6] Ahmed S., Huda M.N., Rajbhandari S., Saha C., Elshaw M., Kanarachos S. (2019). Pedestrian and Cyclist Detection and Intent Estimation for Autonomous Vehicles: A Survey. Appl. Sci..

[7] Kocer B.B., Tjahjowidodo T., Seet G.G.L. (2018). Centralized predictive ceiling interaction control of quadrotor VTOL UAV. Aerosp. Sci. Technol..

[8] Farinha A., Zufferey R., Zheng P., Armanini S.F., Kovac M. (2020). Unmanned Aerial Sensor Placement for Cluttered Environments. IEEE Robot. Autom. Lett..

[9] de Visser E.J., Pak R., Shaw T.H. (2018). From ‘automation’to ‘autonomy’: The importance of trust repair in human—Machine interaction. Ergonomics.

[10] Li S., Zhang X. (2017). Implicit Intention Communication in Human—Robot Interaction Through Visual Behavior Studies. IEEE Trans. Hum. Mach. Syst..

[11] Loukatos D., Arvanitis K.G. (2019). Extending Smart Phone Based Techniques to Provide AI Flavored Interaction with DIY Robots, over Wi-Fi and LoRa interfaces. Educ. Sci..

[12] Fagnant D.J., Kockelman K. (2015). Preparing a nation for autonomous vehicles: Opportunities, barriers and policy recommendations. Transp. Res. Part A Policy Pract..

[13] Blau J. (2015). Apple and google hope to slide into the driver’s seat. Res. Technol. Manag..

[14] Wiesbaden S.F. (2018). Aptiv sends the signal that we are also a mobility provider. ATZelektronik Worldw..

[15] Becker J., Colas M.-B.A., Nordbruch S., Fausten M. (2014). Bosch’s vision and roadmap toward fully autonomous driving. Road Vehicle Automation.

[16] Endsley M.R. (2017). Autonomous Driving Systems: A Preliminary Naturalistic Study of the Tesla Model S. J. Cogn. Eng. Decis. Mak..

[17] Kornhauser A.L., Engineering F., Summary E. (2005). DARPA Urban Challenge Princeton University Technical Paper.

[18] Naujoks F., Wiedemann K., Schömig N., Hergeth S., Keinath A. (2019). Towards guidelines and verification methods for automated vehicle HMIs. Transp. Res. Part F Traffic Psychol. Behav..

[19] Sivak M., Schoettle B. (2016). Would Self-Driving Vehicles Increase Occupant Productivity?.

[20] Choi J.K., Ji Y.G. (2015). Investigating the Importance of Trust on Adopting an Autonomous Vehicle. Int. J. Hum. Comput. Interact..

[21] Fraedrich E., Lenz B., Maurer M., Gerdes J.C., Lenz B., Winner H. (2016). Societal and Individual Acceptance of Autonomous Driving. Autonomous Driving: Technical, Legal and Social Aspects.

[22] Jayaraman S.K., Creech C., Robert L.P., Tilbury D.M., Yang X.J., Pradhan A.K., Tsui K.M. Trust in AV: An Uncertainty Reduction Model of AV-Pedestrian Interactions. Proceedings of the Companion of the 2018 ACM/IEEE International Conference on Human-Robot Interaction.

[23] Marangunić N., Granić A. (2015). Technology acceptance model: A literature review from 1986 to 2013. Univers. Access Inf. Soc..

[24] Wilde G.J.S. (1976). Social Interaction Patterns in Driver Behavior: An Introductory Review. Hum. Factors J. Hum. Factors Ergon. Soc..

[25] Jiang X., Wang W., Bengler K., Guo W. (2015). Analyses of pedestrian behavior on mid-block unsignalized crosswalk comparing Chinese and German cases. Adv. Mech. Eng..

[26] Matú Š. (2014). Road users’ strategies and communication: Driver-pedestrian interaction. Transp. Res. Arena.

[27] Kitazaki S., Myhre N.J. (2015). Effects of Non-Verbal Communication Cues on Decisions and Confidence of Drivers at an Uncontrolled Intersection.

[28] Vissers L., Kint S., Schagen I., Hagenzieker M. (2017). Safe Interaction between Cyclists, Pedestrians and Automated Vehicles: What Do We Know and What Do We Need to Know?.

[29] Fayyad J., Jaradat M.A., Gruyer D., Najjaran H. (2020). Deep Learning Sensor Fusion for Autonomous Vehicle Perception and Localization: A Review. Sensors.

[30] De Clercq K., Dietrich A., Velasco J.P.N., de Winter J., Happee R. (2019). External Human-Machine Interfaces on Automated Vehicles: Effects on Pedestrian Crossing Decisions. Hum. Factors J. Hum. Factors Ergon. Soc..

[31] Kadali B.R., Vedagiri P. (2016). Proactive pedestrian safety evaluation at unprotected mid-block crosswalk locations under mixed traffic conditions. Saf. Sci..

[32] Urmson C.P., Mahon I.J., Dolgov D.A., Zhu J. (2015). Pedestrian Notifications. Google Patents.

[33] Nissan Motor Corporation (2015). Nissan IDS Concept: Nissan’s Vision for the Future of EVs and Autonomous Driving.

[34] Who Sees You When the Car Drives Itself?-SEMCOM. https://semcon.com/smilingcar/.

[35] Clamann M., Aubert M., Cummings M. Evaluation of Vehicle-to-Pedestrian Communication Displays for Autonomous Vehicles. Proceedings of the Transportation Research Board 96th Annual Meeting.

[36] Mahadevan K., Somanath S., Sharlin E. Communicating Awareness and Intent in Autonomous Vehicle-Pedestrian Interaction. Proceedings of the 2018 CHI Conference on Human Factors in Computing Systems.

[37] Böckle M.-P., Brenden A.P., Klingegård M., Habibovic A., Bout M. SAV2P: Exploring the Impact of an Interface for Shared Automated Vehicles on Pedestrians’ Experience. Proceedings of the 9th International Conference on Automotive User Interfaces and Interactive Vehicular Applications Adjunct.

[38] Habibovic A., Andersson J., Lundgren V.M., Klingegård M., Englund C., Larsson S. (2018). External Vehicle Interfaces for Communication with Other Road Users?. Road Vehicle Automation 3.

[39] Ford Motor Corporation (2017). Ford, Virginia Tech Go Undercover to Develop Signals That Enable Autonomous Vehicles to Communicate with People.

[40] Hedlund T., Karlsson A. (2019). Development of an Intuitive Pedestrian Interaction System for Automated Vehicles. Bachelor’s Thesis.

[41] Jandron G.D. (1998). Vehicle Side/Front Brake Lights. Google Patents.

[42] Antonescu O. (2013). Front Stop Lamps for a Safer Traffic. Proceedings of the FISITA 2012 World Automotive Congress.

[43] Petzoldt T., Schleinitz K., Banse R. (2018). Potential safety effects of a frontal brake light for motor vehicles. IET Intell. Transp. Syst..

[44] MB-The F 015 Luxury in Motion Future City. Video. https://www.mercedes-benz.com/en/innovation/autonomous/research-vehicle-f-015-luxury-in-motion/.

[45] Graziano L. AutonoMI Autonomous Mobility Interface. https://vimeo.com/99160686.

[46] Chang C.-M., Toda K., Sakamoto D., Igarashi T. Eyes on a Car: An Interface Design for Communication between an Autonomous Car and a Pedestrian. Proceedings of the 9th International Conference on Automotive User Interfaces and Interactive Vehicular Applications.

[47] Costa G. (2017). Designing Framework for Human-Autonomous Vehicle Interaction. Master’s Thesis.

[48] Deb S., Strawderman L.J., Carruth D.W. (2018). Investigating pedestrian suggestions for external features on fully autonomous vehicles: A virtual reality experiment. Transp. Res. Part. F Traffic Psychol. Behav..

[49] Troel-Madec M., Boissieux L., Borkoswki S., Vaufreydaz D., Alaimo J., Chatagnon S., Spalanzani A. (2019). eHMI Positioning for Autonomous Vehicle/Pedestrians Interaction To Cite this Version: HAL Id: Hal-02388847 eHMI Positioning for Autonomous Vehicle/PEDESTRIANS Interaction.

[50] Fridman L., Mehler B., Xia L., Yang Y., Yvonne L., Bryan F. (2017). To Walk or Not to Walk: Crowdsourced Assessment of External Vehicle-to-Pedestrian Displays. arXiv.

[51] Paolacci G., Chandler J., Ipeirotis P.G. (2010). Running experiments on amazon mechanical turk. Judgm. Decis. Mak..

[52] Dey D., Habibovic A., Pfleging B., Martens M., Terken J. Color and Animation Preferences for a Light Band eHMI in Interactions Between Automated Vehicles and Pedestrians. Proceedings of the 2020 CHI Conference on Human Factors in Computing Systems.

[53] Song Y.E., Lehsing C., Fuest T., Bengler K. External HMIs and their effect on the interaction between pedestrians and automated vehicles. Proceedings of the International Conference on Intelligent Human Systems Integration.

[54] Bazilinskyy P., Dodou D., de Winter J. (2019). Survey on eHMI concepts: The effect of text, color, and perspective. Transp. Res. Part. F Traffic Psychol. Behav..

[55] Ackermann C., Beggiato M., Schubert S., Krems J.F. (2019). An experimental study to investigate design and assessment criteria: What is important for communication between pedestrians and automated vehicles?. Appl. Ergon..

[56] Faas S.M., Mathis L.-A., Baumann M. (2020). External HMI for self-driving vehicles: Which information shall be displayed?. Transp. Res. Part. F Traffic Psychol. Behav..

[57] Chang C.-M., Toda K., Igarashi T., Miyata M., Kobayashi Y. A Video-Based Study Comparing Communication Modalities between an Autonomous Car and a Pedestrian. Proceedings of the Adjunct Proceedings of the 10th International Conference on Automotive User Interfaces and Interactive Vehicular Applications.

[58] Charisi V., Habibovic A., Andersson J., Li J., Evers V. Children’s Views on Identification and Intention Communication of Self-driving Vehicles. Proceedings of the 2017 Conference on Interaction Design and Children.

[59] Li Y., Dikmen M., Hussein T.G., Wang Y., Burns C. To Cross or not to Cross: Urgency-Based External Warning Displays on Autonomous Vehicles to Improve Pedestrian Crossing Safety. Proceedings of the 10th International Conference on Automotive User Interfaces and Interactive Vehicular Applications.

[60] Zhang J., Vinkhuyzen E., Cefkin M. Evaluation of an Autonomous Vehicle External Communication System Concept: A Survey Study. Proceedings of the Advances in Human Factors, Business Management, Training and Education.

[61] Rodriguez P. (2017). Safety of Pedestrians and Cyclists When Interacting with Automated Vehicles—A case study of the WEpods. Master’s Thesis.

[62] Velasco J.P.N., Farah H., van Arem B., Hagenzieker M. WEpod WElly in Delft: Pedestrians’ crossing behavior when interacting with automated vehicles using Virtual Reality. Proceedings of the 15th International Conference on Travel Behavior Research.

[63] Kooijman L., Happee R., De Winter J.C.F. (2019). How Do eHMIs Affect Pedestrians’ Crossing Behavior? A Study Using a Head-Mounted Display Combined with a Motion Suit. Information.

[64] Witmer B.G., Jerome C.J., Singer M.J. (2005). The Factor Structure of the Presence Questionnaire. Presence Teleoperators Virtual Environ..

[65] Otherson I., Conti-Kufner A.S., Dietrich A., Maruhn P., Bengler K. Designing for automated vehicle and pedestrian communication: Perspectives on eHMIs from older and younger persons. Proceedings of the 2018 CHI Conference on Human Factors in Computing Systems.

[66] Colley M., Walch M., Gugenheimer J., Rukzio E. Including People with Impairments from the Start: External Communication of Autonomous Vehicles. Proceedings of the 11th International Conference on Automotive User Interfaces and Interactive Vehicular Applications: Adjunct Proceedings.

[67] Rothenbucher D., Li J., Sirkin D., Mok B., Ju W. Ghost driver: A field study investigating the interaction between pedestrians and driverless vehicles. Proceedings of the 2016 25th IEEE International Symposium on Robot and Human Interactive Communication (RO-MAN).

[68] Huang A.S., Moore D., Antone M., Olson E., Teller S. (2009). Finding multiple lanes in urban road networks with vision and lidar. Auton. Robot..

[69] Lagstrom T., Victor M.L. (2015). AVIP—Autonomous Vehicles’ Interaction with Pedestrians-an investigation of Pedestrian-Driver Communication and Development of a Vehicle External Interface. Master’s Thesis.

[70] Hensch A.-C., Neumann I., Beggiato M., Halama J., Krems J.F. How Should Automated Vehicles Communicate?—Effects of a Light-Based Communication Approach in a Wizard-of-Oz Study. Proceedings of the International Conference on Applied Human Factors and Ergonomics.

[71] Hudson C.R., Deb S., Carruth D.W., McGinley J., Frey D. Pedestrian Perception of Autonomous Vehicles with External Interacting Features. Proceedings of the International Conference on Applied Human Factors and Ergonomics.

[72] Alvarez C., Walter M., Moreno F.M., Sipele O., Smirnov N., Olaverri M. (2020). Autonomous Driving: Framework for Pedestrian Intention Estimationin a Real World Scenario. arXiv.

[73] Colley M., Walch M., Gugenheimer J., Askari A., Rukzio E. Towards Inclusive External Communication of Autonomous Vehicles for Pedestrians with Vision Impairments. Proceedings of the 2020 CHI Conference on Human Factors in Computing Systems.

[74] Rasouli A., Kotseruba I., Tsotsos J.K. Agreeing to cross: How drivers and pedestrians communicate. Proceedings of the 2017 IEEE Intelligent Vehicles Symposium (IV).

[75] ISO 9241-110:2020 Ergonomics of Human-System Interaction—Part 110: Interaction Principles 2020. https://www.iso.org/standard/75258.html.

[76] Jurklies B., Heiligenhaus A., Steuhl K.P., Wessing A. (2016). Human Factors Design Guidance For Driver-Vehicle Interfaces.

[77] SAE Lighting Standard Practices Committee (2016). Color Specification (J578 Ground Vehicle Standard)—SAE Mobilus. Technical Report. SAE. https://saemobilus.sae.org/search/?prodCd=J578.

[78] UNECE (United Nations Economic Commission for Europe) Autonomous Vehicle Signalling Requirements (AVSR) Taskforce. https://wiki.unece.org/pages/viewpage.action?pageId=73925596.

[79] Jansson C., Marlow N., Bristow M. (2004). The influence of colour on visual search times in cluttered environments. J. Mark. Commun..

[80] Brinkley J., Posadas B., Sherman I., Daily S.B., Gilbert J.E. (2019). An Open Road Evaluation of a Self-Driving Vehicle Human–Machine Interface Designed for Visually Impaired Users. Int. J. Hum.-Comput. Interact..

[81] de Leeuw E.D. (2011). Improving Data Quality when Surveying Children and Adolescents: Cognitive and Social Development and its Role in Questionnaire Construction and Pretesting. Report Prepared for the Annual Meeting of the Academy of Finland: Research Programs Public Health Challenges and Health and Welfare of Children and Young People.

